# Socioeconomic status and type 2 diabetes complications among young adult patients in Japan

**DOI:** 10.1371/journal.pone.0176087

**Published:** 2017-04-24

**Authors:** Mitsuhiko Funakoshi, Yasushi Azami, Hisashi Matsumoto, Akemi Ikota, Koichi Ito, Hisashi Okimoto, Nobuaki Shimizu, Fumihiro Tsujimura, Hiroshi Fukuda, Chozi Miyagi, Sayaka Osawa, Ryo Osawa, Jiro Miura

**Affiliations:** 1 Preventive Medicine, Chidoribashi General Hospital, Fukuoka, Japan; 2 Internal Medicine, Jouhoku Hospital, Kanazawa, Japan; 3 Internal Medicine, Kuwamizu Hospital, Kumamoto, Japan; 4 Diabetes Endocrine, Kin-Ikyo Chuo Hospital, Sapporo, Japan; 5 Internal Medicine, Nakano Kyoritsu Hospital, Tokyo, Japan; 6 Diabetes and Metabolism, Saka General Hospital, Shiogama, Japan; 7 Internal Medicine, Kamiina Co-op Hospital, Minowa, Japan; 8 Internal Medicine, Tanaka Clinic, Bihoro, Japan; 9 General Medicine, Juntendo University Hospital, Tokyo, Japan; 10 Endocrine Metabolism, Tachikawa Sogo Hospital, Tachikawa, Japan; 11 General Medicine and Primary Care, University of Tsukuba Hospital, Tsukuba, Japan; 12 Internal Medicine, Kissyouin Hospital, Kyoto, Japan; Universidad Miguel Hernandez de Elche, SPAIN

## Abstract

**Objective:**

To assess the relationship between socioeconomic status (SES) and complications of type 2 diabetes among young adults in Japan.

**Design:**

A cross-sectional study.

**Setting:**

Outpatient wards of 96 member hospitals and clinics of the Japan Federation of Democratic Medical Institutions.

**Participants:**

A total of 782 outpatients with type 2 diabetes (525 males, 257 females), aged 20–40 years as of March 31, 2012. After excluding 110 participants whose retinopathy diagnosis was in question, 672 participants were analyzed.

**Measurements:**

We examined the relations between SES (educational level, income, type of public healthcare insurance, and employment status) and diabetes complications (retinopathy and nephropathy) using a multivariate logistic regression analysis.

**Results:**

The prevalence of type 2 diabetic retinopathy was 23.2%, while that of nephropathy was 8.9%. The odds of having retinopathy were higher among junior high school graduates (OR 1.91, 95% CI 1.09–3.34), patients receiving public assistance (OR 2.19, 95% CI 1.20–3.95), and patients with irregular (OR 1.72, 95% CI 1.03–2.86) or no employment (OR 2.23, 95% CI 1.36–3.68), compared to those with a higher SES, even after covariate adjustment (e.g., age, gender, body mass index). Similarly, the odds of having nephropathy were higher among patients with middle (OR 3.61, 95% CI 1.69–8.27) or low income levels (OR 2.53, 95% CI 1.11–6.07), even after covariate adjustment.

**Conclusions:**

Low SES was associated with a greater likelihood of type 2 diabetes complications in young adults. These findings suggest the necessity of health policies that mitigate socioeconomic disparity and thereby reduce the prevalence of diabetic complications.

## Introduction

Diabetes is a chronic disease with serious complications. Two major complications of T2DM are retinopathy and nephropathy, which are a significant cause of blindness and end-stage renal diseases (ESRD), respectively. In Japan, there are an estimated 9.5 million patients with diabetes—among these, around 15,000 per year will need to begin dialysis due to diabetic ESRD and around 3,000 per year will begin suffering from blindness induced by diabetic retinopathy [1].

Although T2DM can be caused by multiple factors, the most well-known epidemiological risk factors include obesity, physical inactivity, and smoking [2, 3]. However, in recent years, there has been a notable increase in evidence for a relationship between socioeconomic status (SES) and T2DM [2, 4–6]. Nevertheless, there have still been comparatively few investigations of the relationship between SES and T2DM complications [5], and those that have been conducted showed no consistency in terms of the relevance of SES to retinopathy or nephropathy caused by T2DM. T2DM complications are a heavy burden, both for the individuals affected and for society, in terms of quality of life and medical costs. In sum, it is clear that a greater understanding of the relationship between SES and T2DM complications must be obtained.

The majority of T2DM cases are found within adults between the ages of 40 and 50 years [7]; however, obesity among adolescents is rapidly increasing [8], and an increase in T2DM among young adults has been noted [9, 10]. Young adults with T2DM have markedly higher risk of progression to micro- and macrovascular complications compared to middle-aged adults with T2DM [10]. Furthermore, young adults with T2DM appear to be much more likely to develop serious complications, even more so than those with type 1 diabetes of the same age group [11]. Despite this, no report has looked at the relationship between SES and T2DM particularly among young adults. Previous studies on young adults have focused on type 1 diabetes [12]; otherwise, most T2DM research has focused on middle-aged patients [13–16]. Therefore, the relationship between SES and young adults with T2DM is currently unknown.

Due to a long recession, socioeconomic inequality has markedly increased in Japan [17, 18]. Particularly, there has been a drastic increase in the number of irregular employees with low income in Japan, which has resulted in a greater prevalence of young adults with low SES [19]. Individual risk factors such as obesity and physical inactivity remain highlighted as the primary causes of T2DM in Japan; however, almost no study has looked at the relationship between SES and T2DM [20–24] or at that between SES and T2DM complications. Thus, the aims of this study were to identify the potential relationship between SES and T2DM complications among young adult patients.

## Materials and methods

### Participants

The present study was conducted at 96 facilities (53 hospitals and 43 clinics) belonging to the Japan Federation of Democratic Medical Institutions (MIN-IREN). The facilities were located in 38 of the 48 Japanese prefectures, making the sample nationally representative. Participants were 782 outpatients with T2DM who visited these institutions between October 1, 2011 and March 31, 2012; their ages ranged from 20–40 years, as of March 31, 2012. T2DM was diagnosed using the diagnostic criteria of the Japan Diabetes Society’s "Committee report on the classification and diagnostic criteria for diabetes" in 2010 [25]. This allowed us to exclude type 1 diabetes and other types of diabetes such as maturity onset diabetes of the young and gestational diabetes. All patients agreed to participate in the study. Data were collected between June and July 2012 using participants’ medical records and a self-administered questionnaire. After excluding 110 participants whose retinopathy diagnosis was in question, 672 participants were analyzed.

### Measures

#### SES and covariates

Medical records were screened to confirm the diagnosis of T2DM and to obtain data on gender, age, HbA1c (%), presence or absence of diabetic retinopathy and nephropathy, and type of public healthcare insurance. The self-administered questionnaire was used to obtain data on marital status, body mass index (BMI), smoking habits, drinking habits, duration of diabetes, inhabitancy status, physical activity, educational level, income level, and employment status. Patients’ diagnosis of T2DM was made by medical doctors.

In the present study, SES was determined by assessing participants’ education level, income level, type of public healthcare insurance, and employment status. Education level was classified as junior high school, high school, or college (including graduate school). Income level was classified into tertiles of low, middle, and high, which were calculated according to equivalized income (total monthly household income divided by the square root of the numbers of household members). The type of public healthcare insurance—dichotomized as “receiving public assistance” or “another kind of public healthcare insurance”—was used to supplement self-reported income; this was done because public assistance has the advantage of being precisely determined by government authorities and can be easily determined in clinical settings, unlike income. Those who receive public assistance are considered to be at the bottom level of the income distribution. Employment status was classified as regular, irregular, or no employment. Seven students were included in the “no employment” group. In this analysis, junior high school, low income tertile, receiving public assistance, and irregular or no employment were classified as “low SES.” Other options were classified as “high SES.”

Marital status was classified as married or unmarried. The unmarried group included single and divorced patients. BMI classification was set as obese (BMI ≥ 30 kg/m^2^), overweight (BMI ≥ 25 kg/m^2^), or normal (BMI < 25 kg/m^2^). Smoking and drinking history was classified as having experience of smoking/drinking (i.e., being a current or previous smoker/drinker) or no experience of smoking/drinking. As for physical activity, patients were considered to have a regular exercise habit if they exercised for more than 30 minutes twice a week, regardless of the intensity. Inhabitancy status was classified as either living alone or living with others.

#### Diabetes complications

T2DM retinopathy was determined based on a medical doctor’s diagnosis. Furthermore, a patient was considered to have nephropathy if he/she had T2DM retinopathy and his/her proteinuria was found to be high through qualitative testing. This was because high levels of proteinuria are typically regarded as a hallmark of diabetic nephropathy among individuals with diabetic retinopathy [26].

### Statistical analysis

The differences in baseline characteristics according to SES were tested using t-tests (for continuous variables) or chi-square tests (for categorical variables). The relationship between SES and the prevalence of T2DM complications was determined via a multivariate logistic regression analysis while adjusting for demographic factors (age, gender, and marital status), general risk factors (BMI, smoking and drinking experience, and exercise), HbA1C, duration of diabetes, and SES (education level, income level, type of public healthcare insurance, and employment status). Specifically, in Model 1, we adjusted for age and then calculated adjusted odds ratios (ORs) and 95% confidence intervals (95% CIs) for education level, income level, type of public healthcare insurance, and employment status. In Model 2, we adjusted for all the general risk factors as well as demographic factors. We also added a model that included HbA1C and duration of diabetes; as these are considered intermediate variables in the causal chain between SES and T2DM complications, we thought it necessary to investigate them. More specifically, in Model 3, we adjusted for HbA1C and duration of diabetes as well as those factors adjusted for in Model 2. The goodness-of-fit was analyzed using a lack of fit test for every model; the results of this fitness testing indicated that the p-value was not significant for any model, thus indicating that the models had good fit. Importantly, there were missing data in the baseline characteristics. Thus, for every analysis, we configured the software to exclude those participants with missing variables. JMP11 2.1, a statistical analysis program devised by the SAS Institute, Inc., was used for all statistical analyses.

### Ethics statement

The ethics committee at Jouhoku Hospital approved the study and the consent procedure, including information materials and consent forms. All participants provided written informed consent to participate.

## Results

The characteristics of all participants are shown in Table 1. Of all participants, 67.1% were male and 84.5% were in the 30–40 age group. The prevalence of T2DM retinopathy was 23.2%, while that of nephropathy was 8.9%. Concerning SES, 15.6% of participants had completed their junior high school education, the average monthly equivalized household income was 176,000 yen, 9.5% of participants were public assistance recipients, and 27.5% were unemployed; in contrast, the national averages were 5.1% [27], 235,000 yen [28], 0.45–0.76% [29], and 3.1–4.6% [30], respectively. Thus, overall, participants had a lower SES than the national average. Regarding BMI, 44.6% of the total sample was obese.

**Table 1:** General characteristics of patients and prevalence of diabetes complications.

	Total (N = 672)	
	n	%
**Age**		
** 20–29**	104	15.5
** 30–40**	568	84.5
**Gender**		
** Male**	451	67.1
** Female**	221	32.9
**Marital status**		
** Married**	206	31.5
** Single**	413	63.1
** Divorced**	36	5.5
**Education level**		
** Junior high school**	102	15.6
** High school**	316	48.2
** College**	235	35.9
**Income (10**^**4**^ **yen)**		
** Mean (SD)**	583	17.6 (10.5)
**Income level**		
** Low**	175	30.0
** Middle**	201	34.5
** High**	207	35.5
**Public healthcare insurance**		
** Public assistance**	64	9.5
** Other**	608	90.5
**Employment status**		
** No employment**	179	27.5
** Irregular employment**	158	24.3
** Regular employment**	313	48.2
**BMI**		
** Normal**	154	23.5
** Overweight**	209	31.9
** Obese**	292	44.6
**Family history of diabetes**		
** No**	206	32.2
** Yes**	434	67.8
**Drinking experience**		
** Yes (current or former)**	273	41.8
** No**	380	58.2
**Smoking experience**		
** Yes (current or former)**	354	54.0
** No**	301	46.0
**Physical activity**		
** Yes**	181	27.6
** No**	474	72.4
**Inhabitancy status**		
** Alone**	92	14.1
** With others**	563	86.0
**Retinopathy**		
** Yes**	156	23.2
** No**	516	76.8
**Nephropathy**		
** Yes**	60	8.9
** No**	612	91.1
**HbA1c (%)**		
** Mean (SD)**	671	7.7 (1.7)
**Duration of diabetes (years)**		
** Mean (SD)**	649	6.5(5.8)

BMI, body mass index

Table 2 describes the relationship of SES with diabetes risk factors and inhabitancy status. There was a higher prevalence of having alcohol drinking experience among those with regular employment. Furthermore, the prevalence of having smoking experience was higher among those with low education levels and regular workers. Patients who were receiving public assistance were more likely to be living alone.

**Table 2:** General characteristics according to SES (%).

			Education				Income level				Public healthcare insurance			Employment status			
			Junior high school	High school	College	*P*	Low	Middle	High	*P*	Public assistance	Other	*P*	No employment	Irregular employment	Regular employment	*P*
**Total**																	
	**BMI**	**Normal**	25.5	25.0	20.4	N.S.	20.6	26.4	20.3	N.S.	25.8	23.3	N.S.	22.4	26.6	22.7	<0.05
		**Overweight**	27.5	30.7	35.7		33.1	29.4	35.8		24.2	32.7		25.7	38.6	32.3	
		**Obese**	47.1	44.3	43.8		46.3	44.3	44.0		50.0	44.0		52.0	34.8	45.1	
	**Physical activity**	**Yes**	23.5	27.5	29.4	N.S.	31.4	26.4	26.6	N.S.	33.9	27.0	N.S.	33.3	26.0	25.2	N.S.
		**No**	76.5	72.5	70.6		68.6	73.6	73.4		66.1	73.0		66.7	74.1	74.8	
	**Drinking experience**	**Yes (current or former)**	46.1	38.1	44.9	N.S.	39.1	43.3	44.7	N.S.	44.3	41.6	N.S.	28.6	41.1	49.7	<0.001
		**No**	53.9	61.9	55.1		60.9	56.7	55.3		55.7	58.5		71.4	58.9	50.3	
	**Smoking experience**	**Yes (current or former)**	67.7	54.8	47.2	<0.01	48.6	55.7	58.0	N.S.	58.1	53.6	N.S.	44.8	46.8	62.9	<0.001
		**No**	32.4	45.3	52.8		51.4	44.3	42.0		41.9	46.4		55.2	53.2	37.1	
	**Inhabitancy status**	**Living alone**	18.6	13.6	12.8	N.S.	11.4	15.4	15.9	N.S.	41.9	11.1	<0.001	18.0	10.8	13.1	N.S.
		**Living with others**	81.4	86.4	87.2		88.6	84.6	84.1		58.1	88.9		82.0	89.2	86.9	

SES, socioeconomic status; BMI, body mass index

Table 3 shows the adjusted ORs for retinopathy according to SES. In Model 1 (wherein only age was adjusted for), we found that the odds of having retinopathy were greater among junior high school graduates, patients with a low income level, those who were receiving public assistance, and those who had irregular or no employment compared to those with high SES. In Model 2 (after adjusting for general risk factors as well as demographic factors), the odds of having retinopathy were greater among patients who had graduated from junior high school (OR 1.91, 95% CI 1.09–3.34), those who were receiving public assistance (OR 2.19, 95% CI 1.20–3.95), and those who had irregular (OR 1.72, 95% CI 1.03–2.86) or no employment (OR 2.23, 95% CI 1.36–3.68) compared to patients with high SES. Notably, the ORs changed very little after adjusting for the general risk factors. The odds of having retinopathy decreased in Model 3 when compared with Model 2. Although the no employment group included 7 students, similar results (for both the previous and all the following analyses) were obtained after excluding them.

**Table 3:** Multivariate logistic regression analysis for SES and diabetic retinopathy.

	n	Retinopathy (n)	Prevalence of retinopathy (%)	Model 1		Model 2		Model 3	
				OR	95% CI	OR	95% CI	OR	95% CI
**Education level**									
** College**	235	45	19.2	1.00		1.00		1.00	
** High school**	316	76	24.1	1.28	(0.84–1.95)	1.31	(0.86–2.04)	1.29	(0.49–1.22)
** Junior high school**	102	31	30.4	1.85	(1.07–3.17)	1.91	(1.09–3.34)	1.38	(0.75–2.49)
**Income level**									
** High**	207	41	19.8	1.00		1.00		1.00	
** Middle**	201	48	23.9	1.36	(0.84–2.19)	1.44	(0.88–2.36)	1.15	(0.68–1.94)
** Low**	175	47	26.9	1.66	(1.02–2.72)	1.56	(0.94–2.6)	1.12	(0.65–1.94)
**Public healthcare insurance**									
** Others**	608	132	21.7	1.00		1.00		1.00	
** Public assistance**	64	24	37.5	2.27	(1.29–3.92)	2.19	(1.20–3.95)	1.72	(0.91–3.21)
**Employment status**									
** Regular employment**	313	58	18.5	1.00		1.00		1.00	
** Irregular employment**	158	42	26.6	1.80	(1.13–2.85)	1.72	(1.03–2.86)	1.40	(0.82–2.39)
** No employment**	179	52	29.1	2.00	(1.30–3.09)	2.23	(1.36–3.68)	1.71	(1.01–2.90)

OR, odds ratio; 95% CI, 95% confidence interval; SES, socioeconomic status; BMI, body mass index

Model 1: Adjusted for age

Model 2: Model 1 + gender, marital status, BMI, physical activity, smoking, and drinking

Model 3: Model 2 + duration of diabetes and HbA1c

Table 4 shows the ORs for nephropathy based on the SES variables. In Model 2, greater odds of having nephropathy were found among patients who had graduated from junior high school (OR 2.38, 95% CI 1.06–5.31), those with a middle (OR 3.61, 95% CI 1.69–8.27) or low income level (OR 2.53, 95% CI 1.11–6.07), those who were receiving public assistance (OR 2.60, 95% CI 1.16–5.50), and those with irregular (OR 2.83, 95% CI 1.33–6.06) or no employment (OR 4.03, 95% CI 1.95–8.54). The ORs did not decrease after adjusting for general risk factors (i.e., Model 2). The ORs of having nephropathy decreased in Model 3 compared to in Model 2.

**Table 4:** Multivariate logistic regression analysis for SES and diabetic nephropathy.

	n	Nephropathy (n)	Prevalence (%)	Model 1		Model 2		Model 3	
				OR	95% CI	OR	95% CI	OR	95% CI
**Education level**									
** College**	235	15	6.4	1.00		1.00		1.00	
** High school**	316	30	9.5	1.47	(0.78–2.87)	1.56	(0.82–3.01)	1.58	(0.81–3.19)
** Junior high school**	102	14	13.7	2.34	(1.07–5.10)	2.38	(1.06–5.31)	1.71	(0.73–3.95)
**Income level**	207								
** High**	201	11	5.3	1.00		1.00		1.00	
** Middle**	175	25	12.4	2.73	(1.33–5.97)	3.61	(1.69–8.27)	3.05	(1.39–7.16)
** Low**	207	17	9.7	2.15	(0.98–4.89)	2.53	(1.11–6.07)	1.98	(0.84–4.86)
**Public healthcare Insurance**									
** Others**	608	49	8.1	1.00		1.00		1.00	
** Public assistance**	64	11	17.2	2.48	(1.16–4.95)	2.60	(1.16–5.50)	2.11	(0.91–4.61)
**Employment status**									
** Regular employment**	313	19	6.1	1.00		1.00		1.00	
** Irregular employment**	158	18	11.4	2.23	(1.12–4.43)	2.83	(1.33–6.06)	2.34	(1.09–5.07)
** No employment**	183	23	12.5	2.42	(1.27–4.65)	4.03	(1.95–8.54)	3.02	(1.42–6.43)

OR, odds ratio; 95% CI, 95% confidence interval; SES, socioeconomic status; BMI, body mass index

Model 1: Adjusted for age

Model 2: Model 1 + gender, marital status, BMI, physical activity, smoking, and drinking

Model 3: Model 2 + duration of diabetes, HbA1c

## Discussion

### Interpretation of the intermediate variables

We assumed that, in the causal chain model, SES was a cause of T2DM complications via its effects on HbA1C and duration of diabetes. We found that SES was significantly related to both HbA1C and duration of diabetes, and that these two variables were related to T2DM complications (not shown here). In other words, HbA1C and duration of diabetes could be considered intermediate variables, rather than confounding ones, in the causal chain between SES and T2DM complications. In the multivariate analysis, the adjustment of HbA1C and duration of diabetes led to a decrease in the odds of having T2DM complications, which again fit with our hypothesis they are intermediate variables between SES and diabetic complications. Below, we discuss the results in the model without adjustment of HbA1C and duration of diabetes, because the relationships were typically null-biased estimates of the total causal effect after adjusting for the intermediate variables [31].

### Main findings

We examined the relationship between SES and the T2DM complications of retinopathy and nephropathy among young adult outpatients. Our results indicated that among young adult patients with T2DM, the odds of having retinopathy and nephropathy were higher among those with lower SES, such as those who had graduated from junior high school, those with a low or middle income level, those receiving public assistance, and those with irregular or no employment.

### Comparison with past literature

There has been little consistency in past studies regarding the relationship between SES and T2DM complications [13, 16, 32–34]. Our results accord with those of findings on the relation between SES and T2DM prevalence. Furthermore, they accord with studies conducted in Italy and France using the poverty indexes of those countries, which indicated that poorer individuals tended to have a greater prevalence of retinopathy and nephropathy [13, 14]. Similarly, a UK study reported a higher prevalence of retinopathy among those living in deprived areas [15, 16], as did a study in Germany [34]. In contrast, another study in the UK on patients visiting general practitioners found no relationship between the local poverty index and retinopathy or nephropathy [32]. In a retrospective cohort study, also in the UK, no relationship between poverty index and retinopathy was found [33].

In Japan, only four previous studies have discussed the relation of SES with T2DM. A study focusing on public servants found that the prevalence of T2DM was higher among those with low education and low-level work positions [21], whereas a study focusing on white-collar workers reported an increased incidence only among those working in sales jobs [23]. Meanwhile, a study targeting the general population observed that low-income individuals tended to have a higher T2DM treatment rate [22]. The final study, which also looked at the general population, found that the incidence of T2DM was higher among low-income individuals and blue-collar workers [24]. As to the relationship of SES with T2DM complications in Japan, there has been no examination to date; this makes ours the first. As such, further investigation of this relationship in Japan must be performed.

The likely reason for the relation between SES and T2DM complications is that education, income, and occupation are considered social determinants of health; furthermore, as mentioned already, all appear to be related to the prevalence and incidence of T2DM [14, 35–38]. For example, education is considered an index of capacity for practicing a healthy lifestyle and self-management of diseases; individuals with low education levels tend to have higher rates of obesity and unhealthy lifestyles, such as a lack of exercise or alcohol drinking, which are well-known factors influencing the likelihood of diabetes [36]. Previous reports have indicated that up to 45% of the variance in T2DM prevalence by SES can be explained by differences in risk factors such as lack of exercise, obesity, unhealthy eating habits, and smoking behavior [6]. Nevertheless, our study indicated that SES is independently related to T2DM complications, given that the ORs changed very little even after adjusting for general risk factors. Previous studies have also acknowledged that the relationship between SES and T2DM cannot be fully explained by general risk factors [4, 6, 35, 36]. Unfortunately, our results offer no hints as to the mechanism of the relation between SES and T2DM complications. A number of factors are known to mediate the relation between SES and T2DM, such as psychosocial factors (e.g., chronic stress and depression), poor access to health services, and exposure to low SES before birth or during childhood [4, 6, 39, 40]. Thus, it is possible that one such factor—for example, chronic stress—underlies the results of this study.

To our knowledge, this is the first study to examine the relationship between SES and T2DM complications among young adults between the ages of 20 and 40 years. Our results suggest that, as with among middle-aged and older adults, SES is significantly related to T2DM complications among young adults. It seems especially important to recognize this among young adults, given that T2DM complications can easily worsen over time, and that cardiovascular diseases appear to be more prevalent among patients showing an onset of such complications at a younger age than at middle age [10]. Furthermore, psychological stress appears to be higher among young adults with T2DM complications compared to middle-aged and older adults [10, 41]. One previous study indicated the progression of T2DM complications can cause individuals to leave stable jobs, or otherwise can have a negative influence on their occupational life [41]. Overall, it is clear that young adults with T2DM complications have unique problems; thus, further research is necessary to deepen our understanding of the relationship between socioeconomic factors and T2DM among young adults.

### Study implications

Our study indicated that there is an inverse relationship between SES and T2DM complications. T2DM complications lead not only to a decline in patient quality of life, but also to an increase in medical costs, which can eventually impose a greater burden on society. Thus, our results suggest that there is a need for intervention that can improve SES among young adults with T2DM, thereby reducing the odds of them developing T2DM complications, along with other measures to improve healthy behavior [38, 39]. To give some examples, high-quality education among children and adolescents, regardless of their ability to pay, along with ensuring fair and decent employment should be made a central goal of policymaking in Japan. Furthermore, the universal comprehensive social protection might be strengthened [42].

Existing studies on the relationship between SES and T2DM complications were mostly undertaken in Western nations. However, the prevalence and incidence of diabetes and diabetic complications are rapidly increasing in Eastern countries as well, where measures aiming to address these conditions must be enhanced [7, 43]. Our results highlight the necessity of further accumulating studies on the relationship between SES and T2DM complications targeting Asian people, including Japanese.

### Strengths and limitations of the study

This study has several strengths. First, this study provides a comprehensive description of the health characteristics of patients with T2DM and SES among patients with low SES. It is usually rather difficult to obtain such information among individuals with low SES in Japan, as they tend to avoid going to the hospital because of economic reasons [44]. Second, we obtained data with rather high objectivity, because the diagnoses of T2DM and its complications were made by medical doctors. Furthermore, we relied on public healthcare insurance information acquired by medical institutions as a factor of SES in this study; in previous studies on SES and T2DM, diagnoses of T2DM and information on SES were offered by patients in many cases, which can lead to underreporting of chronic conditions, especially among people with low SES [45]. As such, the accuracy of the data on which our results are based is assumed to be high.

Nevertheless, this study also has some limitations. First, the participants tended to be of low SES (i.e., higher rates of junior high school graduates and individuals with low income and receiving public assistance compared to the national average) because of the medical institutions that we sampled. Therefore, it is possible that the prevalence of T2DM retinopathy and nephropathy was overestimated. Second, this was a cross-sectional study; therefore, we cannot exclude the possibility of reverse causality in the findings [37], such as that a patient descends into a low SES as a result of developing a T2DM complication. In other words, developing a T2DM complication might force an individual to shift from regular to irregular or no employment, which can in turn lead them to becoming public assistance recipients due to their reduced income. Third, the sample size is admittedly small, which increases the likelihood of type 2 error. Thus, it would be necessary to conduct further research on the relationship between SES and diabetes complications using a longitudinal design and, if possible, with a larger sample. Fourth, the definition of diabetic nephropathy in this study will likely have led to underestimation of its actual prevalence. We used this definition in order to exclude other kidney diseases characterized by overt proteinuria, such as glomerulonephritis and renal artery stenosis [46]. It is nevertheless unclear whether this definition of diabetic nephropathy would influence the association between SES and T2DM complications. Finally, income had a rather high number of missing values, because it was considered more sensitive personal information compared to the other SES variables. However, the odds of having complications were higher among patients with low or middle income levels, just as they were among those receiving public health assistance (which was also used as an index of lower income). Therefore, the results concerning income seemed reliable, regardless of the number of missing values.

## Conclusions

Our study indicates that low SES—as represented by low education level, low income level, receiving public assistance, and no or irregular employment—is related to a greater prevalence of T2DM complications among young adults. To reduce diabetes complications, it seems necessary to devise an intervention that targets both general risk factors and the social determinants of health. With the growing socioeconomic disparity in Japan, further accumulation of research on the relationship between SES and T2DM is required.

## Supporting information

S1 Table Multiple logistic regression analysis for SES and diabetic retinopathy. (PDF)
