# Supplementary material for: Socioeconomic status and type 2 diabetes complications among young adult patients in Japan
Source: PLoS One. 2017 Apr 24;12(4):e0176087. doi: 10.1371/journal.pone.0176087 (PMC5402943; doi:10.1371/journal.pone.0176087)
Supplement: S1 Table — (PDF) [file pone.0176087.s001.pdf]

S1 Table Multiple logistic regression analysis for SES and diabetic retinopathy

	N	Number of having retinopath y	Prevalenc e(%)	Model 1			Model 2			Model 3		
				OR	95%CI	p–value of goodness–of–fit	OR	95%CI	p–value of goodness–of–fit	OR	95%CI	p–value of goodness–of–fit
Education level												
College	235	45	19.2	1.00			1.00			1.00		
High school	316	76	24.1	1.28	(0.84–1.95)		1.31	(0.86–2.04)		1.29	(0.49–1.22)	
Junior high school	102	31	30.4	1.85	(1.07–3.17)	0.7978	1.91	(1.09–3.34	0.2365	1.38	(0.75–2.49)	0.7322
Income level												
High	207	41	19.8	1.00			1.00			1.00		
Middle	201	48	23.9	1.36	(0.84–2.19)		1.44	(0.88–2.36)		1.15	(0.68–1.94)	
Low	175	47	26.9	1.66	(1.02–2.72)	0.5824	1.56	(0.94–2.61	0.1923	1.12	(0.65–1.94)	0.734
Public healthcare insulance												
Others	608	132	21.7	1.00			1.00			1.00		
Public assistance	64	24	37.5	2.27	(1.29–3.92)	0.1711	2.19	(1.20–3.95	0.2497	1.72	(0.91–3.21)	0.7645
Employment status												
Regular employment	313	58	18.5	1.00			1.00			1.00		
Irregular employment	158	42	26.6	1.80	(1.13–2.85)		1.72	(1.03–2.86)		1.40	(0.82–2.39)	
No employment	179	52	29.1	2.00	(1.30–3.09)	0.9138	2.23	(1.36–3.68	0.2612	1.71	(1.01–2.90)	0.7291

Model 1; Adjusted for age
Model 2; Model 1+gender, marital status,BMI、physical activity、smoking and drinking
Model 3; Model 2 + HbA1C, duration of diabetes
